# The Cu-Li-Sn Phase Diagram: Isopleths, Liquidus Projection and Reaction Scheme

**DOI:** 10.1371/journal.pone.0165058

**Published:** 2016-10-27

**Authors:** Siegfried Fürtauer, Hans Flandorfer

**Affiliations:** Institute of Inorganic Chemistry – Functional Materials, University of Vienna, Währingerstraße 42, A-1090, Vienna, Austria; VIT University, INDIA

## Abstract

The Cu-Li-Sn phase diagram was constructed based on XRD and DTA data of 60 different alloy compositions. Eight ternary phases and 14 binary solid phases form 44 invariant ternary reactions, which are illustrated by a Scheil-Schulz reaction scheme and a liquidus projection. Phase equilibria as a function of concentration and temperature are shown along nine isopleths. This report together with an earlier publication of our group provides for the first time comprehensive investigations of phase equilibria and respective phase diagrams. Most of the phase equilibria could be established based on our experimental results. Only in the Li-rich part where many binary and ternary compounds are present estimations had to be done which are all indicated by dashed lines. A stable ternary miscibility gap could be found which was predicted by modelling the liquid ternary phase in a recent work. The phase diagrams are a crucial input for material databases and thermodynamic optimizations regarding new anode materials for high-power Li-ion batteries.

## Introduction

The combination of *d* elements out of a pool of *n* elements, the number of possibilities *S*, which corresponds to the number of possible phase diagrams, is $\frac{n!}{(n-d)!∙d!}=\frac{65!}{(65-3)!∙3!}$ = 43680 ternary systems. The considered elements *n* exclude nonmetals, noble gases, Tc, elements in 7^th^ period, Ac, Pm and transuranic elements, so *n* = 65; *d* = dimension, unary = 1, binary = 2, ternary = 3,… Nevertheless, only approximately 4000 ternary phase diagrams have been investigated yet [1].

Only few experimental data regarding phase equilibria are available for most of ternary intermetallic systems that contain lithium. The reasons for that are maybe the difficulties and obstacles to prepare and investigate such alloys. This was true for the system Cu-Li-Sn before we started our research which was conducted within the framework of the DFG priority program SPP1473 [2], dedicated to the computational design of new materials for high-power Li-ion batteries. In the meantime, together with our cooperation partners we could establish several new ternary compounds [3–5], four isothermal sections [6], ternary mixing enthalpies [7], and a thermodynamic optimization of the liquid phase [8]. The binary data of Cu-Sn and Li-Sn were taken from recent publications [9, 10], data for Cu-Li refer to an earlier work [11].

This work is not only a fundamental description of the new ternary system Cu-Li-Sn, but also gives insights into equilibrium states for possible materials for the tailored design of Li-ion battery anodes. Improved cell design using well-established materials will not be sufficient for a mandatory enhancement of energy and power density, and thus new materials have to be found. Advanced anode materials, *e*.*g*. intermetallics, are suggested for the use in such battery applications.

Despite battery performance testing of Cu-Sn alloy anodes, performed by several authors [12–15] who have proposed mechanisms for the lithiation of η´-Cu_6_Sn_5_, the understanding of these processes is scarce without detailed knowledge of involved phases, their equilibria and structures. Although, information on equilibrium states is not sufficient to understand and predict battery performance, which is highly influenced by kinetics, phase diagrams are fundamental.

This work on Cu-Li-Sn phase relations together with isothermal sections recently published by the same authors [6] and experimental thermochemical data [7, 8] provides thermodynamic information necessary for a comprehensive assessment and optimization of the respective phase diagram using CALPHAD methods.

## Experimental Procedure

### Sample preparation

**Fig 1:**
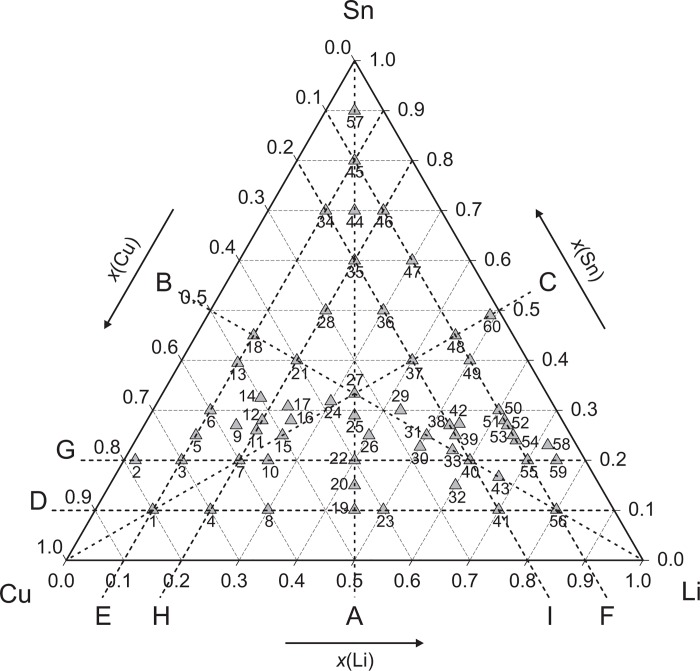
Sample compositions and isopleth sections (A: *x*_Cu_: *x*_Li_ = 0.5; B: *x*_Cu_: *x*_Sn_ = 0.5; C: *x*_Li_: *x*_Sn_ = 0.5; D: *x*_Sn_ = 0.1; E: *x*_Li_ = 0.1; F: *x*_Cu_ = 0.1; G: *x*_Sn_ = 0.2; H: *x*_Li_ = 0.2; I: *x*_Cu_ = 0.2).

Intermetallic samples, which are located mainly along nine sections across the Gibbs triangle, have been prepared at 60 different compositions from pure elements Cu (99.98 wt. %, wire, Goodfellow, Cambridge, UK), Li (99.8 wt. %, wire, Alfa Aesar, Karlsruhe, Germany) and Sn (99.95 wt. %, ingot, Advent, Oxford, UK). The sample compositions are shown in Fig 1 together with the nine cross sections. The Cu-wire was treated in a H_2_-flow for 5 hours at 300°C to remove the natural thin oxide layer at the surface. The Li-wire, which was stored originally in mineral oil for oxidation prevention, was cleaned by n-hexane in a supersonic bath. Visible oxidations spots were scraped off with a knife. All manipulations with Li or Li-containing samples were performed in a glove box under Ar atmosphere (< 5 ppm O_2_ / H_2_O). Samples have been weighed in thimble-like Ta crucibles, which have been welded with a corresponding lid in an arc furnace. For melting the enclosed metals, the crucibles were put into an induction furnace at 1100°C. Repetition of the melting process twice (only 10–20 sec. each to prevent high temperature fatigue of the welding seam) with turning the crucible upside down between the heating steps assured homogenous mixing of the liquid alloys. Then the crucibles were sealed in quartz glass tubes under vacuum. All alloys were annealed consequently in a muffle furnace at 400°C for several weeks and subsequently at 300, 400, 500 and 600°C; few of them were annealed also at other temperatures (see Table 1). Especially in case of samples with high Li-contents the Ta sheet became partially permeable for Li vapour. This was evidenced by a darkening of the surrounding quartz glass, which could be explained by a reduction of transparent SiO_2_ to brown SiO or related Li-containing silicates. After the heat treatment, all samples were quenched in cold water and checked for mass loss. In most cases, the mass loss was negligible with respect to the extension of the respective phase fields.

**Table 1:** Heat treatment and quenched phases of Cu-Li-Sn samples at annealing temperatures.

*x*(Cu)^1^	*x*(Li)^1^	*x*(Sn)^1^	Sample No.	Shown in isopleth	Heat treatment	Identified phases^2^	Corresponding phase field^3^	Formed during quenching	Formed during hydrolysis	Comments
0.80	0.10	0.10	1	C, D, E	400°C / 27d	(Cu), T2, T1*	(Cu), T1, T2			
0.78	0.02	0.20	2	G	400°C / 65d	ζ, T2, (Cu)*	(Cu), δ, T2			ζ and δ very similar in XRD
0.78	0.02	0.20	2	G	600°C / 91d	ζ, δ, T2*	D0_3_-γ, δ, T2	ζ (from D0_3_-γ)		
0.70	0.10	0.20	3	E, G	400°C / 9d	ζ, T2, (Cu)	(Cu), δ, T2			ζ and δ very similar in XRD
0.70	0.10	0.20	3	E, G	700°C / 22d	ζ, T2, ε	Liq, β, T2	ε, ζ (from Liq, β)		
0.70	0.20	0.10	4	D, H	400°C / 27d	(Cu), T1	(Cu), T1			
0.70	0.20	0.10	4	D, H	600°C / 3d	(Cu), T1	(Cu), T1			
0.65	0.10	0.25	5	E	400°C / 27d	ε, T3, T2*	ε, T2, T3			
0.65	0.10	0.25	5	E	500°C / 100d	ε, T3, T2*	Liq, ε, T2	T3		
0.65	0.10	0.25	5	E	600°C / 100d	ε, T2, ζ*, T4*	ε, ζ, T2	T4*		T4 from quenched liquid (minor amount)
0.65	0.10	0.25	5	E	700°C / 100d	ζ, ε, T2	Liq, β, T2	ε, ζ (from Liq, β)		
0.60	0.10	0.30	6	E	300°C / 105d	ε, T3, η	ε, η, T3			
0.60	0.10	0.30	6	E	400°C / 70d	T3, ε, η, (Sn)*, T2*	Liq, ε, T3	(Sn), η, T2		
0.60	0.10	0.30	6	E	500°C / 65d	ε, T3, η*, (Sn)*	Liq, ε, T2	(Sn), η, T3		Composition shifted to Liq + ε
0.60	0.10	0.30	6	E	600°C / 65d	ε, T3, η, (Sn)*	Liq, ε, T2	(Sn), η, T3		Composition shifted to Liq + ε
0.60	0.20	0.20	7	C, G, H	400°C / 21d	T2, (Cu)	T2, (Cu)			
0.60	0.30	0.10	8	D	400°C / 9d	(Cu), T5, T1	(Cu), T1, T5			
0.60	0.30	0.10	8	D	500°C / 7d	(Cu), T5, T1	(Cu), T1, T5			
0.57	0.16	0.27	9	-	400°C / 65d	T2, T3, ε	ε, T2, T3			No isopleth
0.57	0.16	0.27	9	-	600°C / 91d	T2, ζ, ε, η*, T3*, T4*, (Sn)*	Liq, ε, T2	(Sn), ζ, η, T3, T4		No isopleth
0.55	0.25	0.20	10	G	400°C / 70d	T1, T2, (Cu)	(Cu), T1, T2			
0.54	0.20	0.26	11	H	500°C / 35d	T2, T3*	Liq, T2	T3		
0.52	0.20	0.28	12	H	400°C / 21d	T3, T2, ε	ε, T2, T3			
0.52	0.20	0.28	12	H	500°C / 35d	T2, δ, T3*, T4*, (Sn)*, η*	Liq, T2	(Sn)*, δ, η*, T3*, T4*		
0.51	0.10	0.40	13	E	300°C / 122d	η, T3, ε	ε, η, T3			
0.51	0.10	0.40	13	E	400°C / 21d	ε, T3, T4*, T2*, η*, (Sn)*	Liq, ε, T3	T4*, T2*, η*, (Sn)*		
0.51	0.10	0.40	13	E	500°C / 35d	ε, η, (Sn), T4*	Liq, ε	(Sn), η, T4*		
0.51	0.10	0.40	13	E	600°C / 35d	ε, η, T3, (Sn)	Liq, ε	(Sn), η, T3		
0.50	0.18	0.33	14	-	300°C / 105d	T3, ε, η, (Sn)*	ε, η, T3		(Sn)*	No isopleth
0.50	0.18	0.33	14	-	400°C / 70d	T3, ε, η, T2*, (Sn)*	Liq, ε, T3	(Sn)*, η, T2*		No isopleth
0.50	0.18	0.33	14	-	500°C / 65d	T2, δ, ε, T3, η, (Sn)	Liq, ε, T2	(Sn), δ, η, T3		No isopleth
0.50	0.25	0.25	15	C	300°C / 84d	T2	T2			
0.50	0.25	0.25	15	C	400°C / 35d	T2	T2			
0.50	0.25	0.25	15	C	600°C / 84d	T2	T2			
0.47	0.25	0.28	16	-	400°C / 21d	T2, T3, T4, (Sn)*	T2, T3, T4		(Sn)*	No isopleth
0.47	0.25	0.28	16	-	500°C / 27d	T2, η, (Sn), T4*	Liq, T2	(Sn), η, T4*		No isopleth
0.47	0.25	0.28	16	-	600°C / 35d	T2, T4, η*, (Sn)*	Liq, T2	(Sn)*, η*, T4		No isopleth
0.46	0.23	0.31	17	-	400°C / 70d	T3, T4*	T3, T4			No isopleth
0.46	0.23	0.31	17	-	500°C / 65d	T2, δ, (Sn)*, η*	Liq, T2	(Sn)*, δ, η*		No isopleth
0.45	0.10	0.45	18	B, E	300°C / 100d	η, T4, (Sn)	Liq, η, T4	(Sn)		
0.45	0.10	0.45	18	B, E	400°C / 27d	ε, T3, (Sn), η	Liq, ε, T3	(Sn), η		
0.45	0.45	0.10	19	A, D	400°C / 27d	(Cu), Li_17_Sn_4_, (Sn)*	(Cu), Li_17_Sn_4_		(Sn)*	
0.43	0.43	0.15	20	A	400°C / 27d	T5, (Cu), T1	(Cu), T1, T5			
0.40	0.20	0.40	21	B, H	300°C / 100d	T4, η, T3, (Sn)*	η, T3, T4		(Sn)*	
0.40	0.20	0.40	21	B, H	400°C / 27d	T3, η, (Sn), ε*, T4*	Liq, ε, T3	(Sn), η, T4*		
0.40	0.20	0.40	21	B, H	500°C / 100d	T2, T4, η, T3*, (Sn)*	Liq, T2	(Sn)*, η, T3*, T4		
0.40	0.40	0.20	22	A, G	400°C / 56d	T1, (Cu)	(Cu), T1			
0.40	0.40	0.20	22	A, G	600°C / 3d	T1, (Cu)	(Cu), T1			
0.40	0.50	0.10	23	D	400°C / 9d	(Cu), Li_17_Sn_4_, T5, (Sn)*	Liq, (Cu), Li_17_Sn_4_	(Sn)*, T5		
0.40	0.50	0.10	23	D	650°C / 2d	(Cu), T5, Li_17_Sn_4_	Liq, (Cu), Li_17_Sn_4_	T5		
0.40	0.50	0.10	23	D	750°C / 4d	Li_17_Sn_4_, T5, (Cu)	Liq, (Cu), Li_17_Sn_4_	T5		
0.38	0.30	0.32	24	-	400°C / 42d	T3, T4, (Sn)*	T3, T4		(Sn)*	No isopleth
0.38	0.30	0.32	24	-	500°C / 77d	T2, T4, (Sn)*	Liq, T2, T4	(Sn)*		No isopleth
0.38	0.30	0.32	24	-	600°C / 77d	T2, T4, η, (Sn)	Liq, T2	(Sn), η, T4		No isopleth
0.36	0.36	0.29	25	A	400°C / 21d	T1, T4, η*, T2*, (Sn)*	T1, T2, T4,		(Sn)*, η*	
0.36	0.36	0.29	25	A	650°C / 4d	T1, T2, T4, η, (Sn)*	Liq, T1, T2	(Sn)*, η, T4		
0.35	0.40	0.25	26	-	400°C / 70d	T1	T1			No isopleth
0.33	0.33	0.33	27	A, B, C	400°C / 70d	T4, T2, T3	T2, T3, T4			
0.33	0.33	0.33	27	A, B, C	500°C / 65d	T2, T4, η, (Sn)	Liq, T2, T4	(Sn), η		
0.33	0.33	0.33	27	A, B, C	600°C / 65d	T2, η, (Sn), T4, T1*	Liq, T1, T2	(Sn), η, T4		
0.30	0.20	0.50	28	H	300°C / 77d	η, T4, (Sn)	Liq, η, T4	(Sn)		
0.30	0.20	0.50	28	H	400°C / 9d	T3, η, (Sn), ε*	Liq, ε, T3	(Sn), η		
0.27	0.43	0.30	29	-	400°C / 70d	T4, T1	T1, T4			No isopleth
0.27	0.43	0.30	29	-	650°C / 3d	T1, T4, η, (Sn)*	Liq, T1	(Sn)*, η, T4		No isopleth
0.27	0.50	0.23	30	-	400°C / 42d	T1, T5, (Cu)	(Cu), T1, T5			No isopleth
0.25	0.50	0.25	31	B	400°C / 35d	T1	T1			
0.25	0.50	0.25	31	B	600°C / 4d	T1, T6, T4	T1, T6	T4		Composition shifted to Liq + T1 + T6
0.25	0.60	0.15	32	-	400°C / 56d	(Cu), Li_17_Sn_4_, T5	(Cu), Li_17_Sn_4_, T5			No isopleth
0.22	0.56	0.22	33	B	400°C / 54d	T5, T1, (Cu)	(Cu), T1, T5			
0.22	0.56	0.22	33	B	600°C / 2d	T5, T1, η, (Sn)*, (Cu)*	(Cu), T1, T5		(Sn)*, η	
0.20	0.10	0.70	34	E, I	300°C / 100d	(Sn), η, T4	Liq, η, T4	(Sn)		
0.20	0.10	0.70	34	E, I	400°C / 27d	(Sn), η, T4	Liq	(Sn), η, T4		
0.20	0.20	0.60	35	A, H, I	300°C / 100d	T4, η, (Sn)	Liq, η, T4	(Sn)		
0.20	0.20	0.60	35	A, H, I	400°C / 27d	T3, T4, η, (Sn), ε*	Liq, ε, T3	(Sn), η, T4		
0.20	0.30	0.50	36	I	300°C / 105d	T4, η, (Sn)	Liq, η, T4	(Sn)		
0.20	0.30	0.50	36	I	400°C / 70d	T4, T3, η*, (Sn)*	Liq, T3, T4	(Sn)*, η*		
0.20	0.30	0.50	36	I	500°C / 65d	T4, (Sn), η	Liq, T4	(Sn), η		
0.20	0.40	0.40	37	C, I	300°C / 77d	T4, η, (Sn)	Liq, T4	(Sn), η		
0.20	0.40	0.40	37	C, I	400°C / 70d	T4, η, (Sn)	Liq, T4	(Sn), η		
0.20	0.40	0.40	37	C, I	500°C / 77d	T4, η, (Sn)	Liq, T4	(Sn), η		
0.20	0.53	0.27	38	I	400°C / 65d	T6, T1	T6, T7, T8			Composition shifted to T1 + T6
0.20	0.53	0.27	38	-	650°C / 5d	T6, T1	T6, T7		Phase field shifted to T1 + T6	
0.20	0.55	0.25	39	I	400°C / 27d	T1, T6, η, (Sn)*	T1, T6, T7	(Sn)*, η		Composition shifted to Liq + T1 + T6
0.20	0.55	0.25	39	I	600°C / 3d	T1, T6, η, (Sn)*	T1, T6, T7	(Sn)*, η		Composition shifted to Liq + T1 + T6
0.20	0.60	0.20	40	B, G, I	400°C / 27d	T5, (Cu)	T5, (Cu)			
0.20	0.70	0.10	41	D, I	400°C / 9d	Li_17_Sn_4_, (Cu), (Li)	Liq, (Cu), Li_17_Sn_4_	(Li)		
0.20	0.70	0.10	41	D, I	600°C / 2d	(Li), Li_17_Sn_4_, (Cu)	Liq, (Cu), Li_17_Sn_4_	(Li)		
0.18	0.55	0.27	42	-	400°C / 65d	T1, T6, Li_13_Sn_5_*, (Sn)*	Li_13_Sn_5_, T6, T8		(Sn)*	No isopleth, composition shifted to Li_13_Sn_5_ + T1 + T6
0.17	0.67	0.17	43	B	400°C / 21d	Li_17_Sn_4_, (Cu), T5	(Cu), Li_17_Sn_4_, T5			
0.15	0.15	0.70	44	A	200°C / 60d	T4, η, (Sn)	(Sn), η, T4			
0.10	0.10	0.80	45	A, E, F	200°C / 60d	η, T4, (Sn)	(Sn), η, T4			
0.10	0.20	0.70	46	F, H	300°C / 100d	T4, (Sn), η	Liq, η, T4	(Sn)		
0.10	0.20	0.70	46	F, H	400°C / 27d	(Sn), η, T4	Liq	(Sn), η, T4		
0.10	0.30	0.60	47	F	300°C / 77d	T4, (Sn), Li_2_Sn_5_, η	Liq, Li_2_Sn_5_, T4	(Sn), η		
0.10	0.30	0.60	47	F	400°C / 9d	T4, (Sn), Li_2_Sn_5_, η	Liq, T4	(Sn), η, Li_2_Sn_5_		
0.10	0.45	0.45	48	C, F	300°C / 100d	T4, LiSn, Li_2_Sn_5_*, η*, (Sn)*	LiSn, Li_2_Sn_5_, T4		(Sn)*, η*	
0.10	0.45	0.45	48	C, F	400°C / 27d	T4, η, (Sn), LiSn, Li_2_Sn_5_*, Li_7_Sn_3_*, T1*	Liq, LiSn, T4	(Sn), η, Li_2_Sn_5_*, Li_7_Sn_3_*, T1*		
0.10	0.45	0.45	48	C, F	500°C / 100d	T4, LiSn, eta*, Li_2_Sn_5_*, (Sn)*	Liq, Li_7_Sn_3_, T6	LiSn, eta*, Li_2_Sn_5_*, (Sn)*		Composition shifted to Liq + T4
0.10	0.50	0.40	49	F	400°C / 9d	T1, LiSn, (Sn), η, Li_7_Sn_3_	LiSn, Li_7_Sn_3_, T1	(Sn), η		(Sn), η from quenched liquid (minor amount)
0.10	0.50	0.40	49	F	500°C / 22d	T4, (Sn), Li_7_Sn_3_, T6*	Liq, Li_7_Sn_3_, T6	(Sn), T4		
0.10	0.60	0.30	50	F	400°C / 65d	T1, Li_7_Sn_3_, Li_13_Sn_5_, (Sn)*	Li_7_Sn_3_, T1	(Sn)*, Li_13_Sn_5_		(Sn)*, Li_13_Sn_5_ from quenched liquid (minor amount)
0.10	0.60	0.30	50	F	600°C / 5d	Li_7_Sn_3_, T1, T4	Liq, Li_5_Sn_2_, T6	Li_7_Sn_3_, T4		Composition shifted to Liq + T1
0.10	0.62	0.28	51	F	400°C / 21d	Li_13_Sn_5_, T1, Li_5_Sn_2_	Li_5_Sn_2_, Li_13_Sn_5_, T1			
0.10	0.62	0.28	51	-	650°C / 3h	T1, Li_13_Sn_5_, (Sn)*	Liq, Li_13_Sn_5_, T8		(Sn)*	Phase field shifted to T1 + Li_13_Sn_5_
0.10	0.63	0.27	52	F	400°C / 7d	T6, Li_13_Sn_5_	Li_13_Sn_5_, T6, T8			Composition shifted to Li_13_Sn_5_ + T6 + T8
0.10	0.65	0.25	53	F	400°C / 34d	T6, T1, Li_13_Sn_5_*, (Sn)*	Li_13_Sn_5_, T6, T8	(Sn)*, T1		Composition shifted to Li_13_Sn_5_ + T1 + T6
0.10	0.65	0.25	53	F	600°C / 2d	T6, T4, T1, (Sn)*	Li_13_Sn_5_, T6, T8	(Sn)*, T1, T4		Composition shifted to Liq + T1 + T6
0.10	0.66	0.24	54	F	400°C / 21d	T6, T1, T8, (Sn)*	Li_13_Sn_5_, T8			Composition shifted to Li_13_Sn_5_ + T8
0.10	0.66	0.24	54	F	600°C / 2d	T6, T1, T8, (Sn)*	Li_7_Sn_2_, Li_13_Sn_5_, T8			Composition shifted to Li_7_Sn_2_ + Li_13_Sn_5_ + T8
0.10	0.70	0.20	55	G, F	400°C / 9d	(Sn), T7	Li_7_Sn_2_, Li_17_Sn_4_, T5			Composition shifted to Li_7_Sn_2_ + Li_17_Sn_4_ + T5
0.10	0.70	0.20	55	-	650°C / 4d	T1, T5, T4, (Sn)*	Li_17_Sn_4_, T5	T4, (Sn)*		Phase field shifted toLiq + T1 + T5
0.10	0.80	0.10	56	B, D, F	400°C / 9d	Li_17_Sn_4_, (Cu)	Liq, (Cu), Li_17_Sn_4_	(Li)		(Li) hardly visible in XRD
0.10	0.80	0.10	56	B, D, F	600°C / 2d	Li_17_Sn_4_, (Cu)	Liq, (Cu), Li_17_Sn_4_	(Li)		(Li) hardly visible in XRD
0.05	0.05	0.90	57	A	200°C / 60d	(Sn), η	(Sn), η, T4			Very low amount of T4
0.05	0.72	0.23	58	-	400°C / 21d	Li_7_Sn_2_, Li_17_Sn_4_, T1	Li_7_Sn_2_, T7	T1, T4*, T5		No isopleth, composition shifted to Li_7_Sn_2_ + Li_17_Sn_4_ + T1
0.05	0.75	0.20	59	G	400°C / 14d	T5, Li_17_Sn_4_	Li_17_Sn_4_, T5			
0.02	0.49	0.49	60	C	300°C / 91d	LiSn, T4, Li_2_Sn_5_, (Sn)*	LiSn, Li_2_Sn_5_, T4		(Sn)*	
0.02	0.49	0.49	60	C	400°C / 65d	LiSn, Li_2_Sn_5_, (Sn), η*, T4*	Liq, LiSn, T4	(Sn), η*, Li_2_Sn_5_		

^1^ Samples are ordered with decreasing Cu- and increasing Li-concentration

^2^ Identified phases are ordered with decreasing amount (approximated from Rietveld-refinements)

^3^ Corresponding phase fields are ordered systematically (Liq – unary – binary – ternary phases)

* denotes phases, which are only present in traces.

### Analytical method: Powder XRD

Ta crucibles were opened in the glove box with a bolt cutter and the alloys have been extracted by squeezing the crucible. The (usually) brittle samples were powdered with a Durit® mortar and fixed with petroleum jelly on a specimen holder consisting of a silicon monocrystal. It was covered with a gastight polycarbonate cap before shuttled out of the glove box. Samples were exposed to Cu-K_α_ X-ray radiation (40 kV / 40 mA) in a diffractometer equipped with Bragg-Brentano geometry and a Ni filter. Signals were detected by a strip detector. Full-profile Rietveld refinements were applied for phase analyses which are presented in Table 1. Crystallographic information of the binary and ternary phases were listed in [6] recently.

### Analytical method: DTA

Approximately 100–150 mg of sample material, which was annealed at 400°C in order to establish starting equilibrium condition, was filled in Ta crucibles with a flattened bottom. The crucibles were closed with a corresponding lid and welded with an arc furnace. Thermal analysis was done in a single-point DTA instrument, equipped with small alumina discs as spacers between the crucible bottom and the welding bead of the S-type thermocouple. Reference material was a comparable amount of alumina in a second Ta crucible. Temperature calibration was done with pure metals, as Sn, Ag and Au, as well enclosed in Ta crucibles. The furnace program for the measurements was as follows: Fast heating to annealing temperature (20°C / min up to 400°C)—equilibration for 30 min—heating with 5°C / min until 50 K above the estimated liquidus temperature (however, < 900°C to prevent leakage of the crucibles) – cooling with 5°C / min to 100°C – second heating with 5°C / min to estimated liquidus temperature – cooling with 5°C / min to room temperature. Peaks were evaluated with the Netzsch Proteus® software [16], overlapping peaks have been separated by the peak deconvolution tool in the Calisto® software package from AKTS [17]. Characteristic temperatures were determined by evaluation of the peak onset of the respective DTA signals on heating—except liquidus temperatures which correspond to the peak maximum (see Table 2). The estimated error of the temperature measurement is ± 2 K what is relatively high and attributed to the use of Ta crucibles.

**Table 2:** DTA results of all samples.

*x*(Cu)	*x*(Li)	*x*(Sn)	Sample No.	Shown in isopleth	Heat Treatment [°C]	Thermal effects during heating [°C]	Liq. [°C]
0.80	0.10	0.10	1	C, D, E	27d / 400°C	745; 747	891
0.78	0.02	0.20	2	G	65d / 400°C	*534*; 734	741
0.70	0.10	0.20	3	E, G	9d / 400°C	686; 735; 747	751
0.70	0.20	0.10	4	D, H	27d / 400°C	725; 738	*
0.65	0.10	0.25	5	E	27d / 400°C	442; *508; 541; 663*; *674;* 730	743
0.60	0.10	0.30	6	E	70d / 400°C	*209; 325*; 349; 445; *505*; 634; 687	714
0.60	0.20	0.20	7	C, G, H	21d / 400°C	744	*
0.60	0.30	0.10	8	D	9d / 400°C	447; 729	*
0.57	0.16	0.27	9	-	65d / 400°C	215; 436; 738	752
0.55	0.25	0.20	10	G	70d / 400°C	745	*
0.54	0.20	0.26	11	H	21d / 400°C	456; 754	763
0.52	0.20	0.28	12	H	21d / 400°C	*422*; 444; 720	752
0.51	0.10	0.40	13	E	21d / 400°C	*211; 328;* 349; 447; *504;* 524	645
0.50	0.18	0.33	14	-	70d / 400°C	211; 326; 446; 455; 653	710
0.50	0.25	0.25	15	C	35d / 400°C	*531*; 763	774
0.47	0.25	0.28	16	-	21d / 400°C	457; 549; 727	753
0.46	0.23	0.31	17	-	70d / 400°C	210; 423; 461; 485; 588; 683	735
0.45	0.10	0.45	18	B, E	27d / 400°C	216; 332; 346; *527*	582
0.45	0.45	0.10	19	A, D	27d / 400°C	727; 812	828
0.43	0.43	0.15	20	A	27d / 400°C	730; 804	833
0.40	0.20	0.40	21	B, H	27d / 400°C	*219*; 338; 451; 460;	651
0.40	0.40	0.20	22	A, G	56d / 400°C	*523;* 720; 730	*
0.40	0.50	0.10	23	D	9d / 400°C	*629; 668; 693*; 827	837
0.38	0.30	0.32	24	-	42d / 400°C	426; 463; 474; 550; 694	722
0.36	0.36	0.29	25	A	21d / 400°C	594; *740*	757
0.35	0.40	0.25	26	-	70d / 400°C	759	770
0.33	0.33	0.33	27	A, B, C	70d / 400°C	459; *552*; 570; 670	719
0.30	0.20	0.50	28	H	9d / 400°C	187; 336; 348; 454; 485	538
0.27	0.43	0.30	29	-	70d / 400°C	599; 709	746
0.27	0.50	0.23	30	-	42d / 400°C	-	-
0.25	0.50	0.25	31	B	35d / 400°C	*529*; 690; 721	750
0.25	0.60	0.15	32	-	56d / 400°C	709; 713; 788	813
0.22	0.56	0.22	33	B	54d / 400°C	*469*; 717	729
0.20	0.10	0.70	34	E, I	27d / 400°C	216; ~318; ~370	~380
0.20	0.20	0.60	35	A, H, I	27d / 400°C	218; 344; 422	450
0.20	0.30	0.50	36	I	70d / 400°C	212; 330; *431*; 455; 471	530
0.20	0.40	0.40	37	C, I	9d / 400°C	217; 346; 457; 584; 606	632
0.20	0.53	0.27	38	I	65d / 400°C	*619*; 660; 707	739
0.20	0.55	0.25	39	I	27d / 400°C	*520*; 697; 708; 709	736
0.20	0.60	0.20	40	B, G, I	27d / 400°C	736; 779	782
0.20	0.70	0.10	41	D, I	9d / 400°C	180; *639; 662; 679*; 687	712
0.18	0.55	0.27	42	-	65d / 400°C	609; 618; 671; 698	731
0.17	0.67	0.17	43	B	21d / 400°C	*708*; 732; 837	839
0.15	0.15	0.70	44	A	60d / 400°C	219	~430
0.10	0.10	0.80	45	A, E, F	60d / 400°C	221	~350
0.10	0.20	0.70	46	F, H	27d / 400°C	215; ~380	~390
0.10	0.30	0.60	47	F	9d / 400°C	218; *299*; 431	471
0.10	0.45	0.45	48	C, F	27d / 400°C	323; 475; 503; 560	586
0.10	0.50	0.40	49	F	9d / 400°C	470; 584	599
0.10	0.60	0.30	50	F	65d / 400°C	507; *589; 596*; 615; 646	678
0.10	0.62	0.28	51	F	21d / 400°C	618; 645; 681	695
0.10	0.63	0.27	52	F	7d / 400°C	688	697
0.10	0.65	0.25	53	F	34d / 400°C	*482*; 690; 700; 709	718
0.10	0.66	0.24	54	F	21d / 400°C	500; 675; 693; 703; 713	721
0.10	0.70	0.20	55	F, G	9d / 400°C	*490; 603*; 695; 705; 711	723
0.10	0.80	0.10	56	B, D, F	9d / 400°C	180; 581; 681	685
0.05	0.05	0.90	57	A	60d / 400°C	222	~330
0.05	0.72	0.23	58	-	21d / 400°C	703; 720; 728	752
0.05	0.75	0.20	59	G	14d / 400°C	718	727
0.02	0.49	0.49	60	C	65d / 400°C	321; 478	484

Thermal effects, which could not directly be considered in isopleths, are in *italic letters*.

*Liquidus temperature was not reached

## Results and Discussion

The present work visualizes the Cu-Li-Sn phase diagram, which was constructed based on experimental data. Since there are no such equilibrium phase diagrams for the Cu-Li-Sn system available in literature, it is the first comprehensive description of phase relations for all compositions and temperatures up to 1200°C and at atmospheric pressure. It is in consistence with four ternary isothermal sections at temperatures between 300 and 600°C which have been recently published by the authors [6] and considers recent findings of new ternary intermetallic compounds [3–5, 18] and the binary subsystems [8–10, 19]. There is strong experimental evidence for a stable liquid miscibility gap in the ternary system, which is discussed in more detail below.

### XRD data

60 alloy samples with different compositions have been prepared and annealed at 400°C and in some cases at various other temperatures. At all 122 samples have been investigated by XRD and the results are listed in Table 1. In most cases, the phase analysis showed a consistent picture. Some samples have been annealed between 650 and 750°C (see Table 1) to check the presence of liquid phase at the respective temperature. This is indicated by the occurrence of non-equilibrium phases from the solidification of liquid phase during quenching. It was the case for samples 23, 25, 51 (annealed at 650°C), samples 3, 5 (annealed at 700°C) and sample 23 annealed at 750°C. The detected equilibrium phases of samples 38, 51 and 55 (all annealed at 650°C) are in contradiction to the phase equilibria, which have been deduced from several other samples. They seem to be shifted towards lower Li-concentrations. This might be caused by Li-losses during sample preparation or by inhomogeneities. Therefore, these samples were not included into the respective isopleths. Samples 44 and 45, both annealed at 200°C, contain the phases (Sn) + η + T4. Sample 57 is as well allocated to this three-phase field, and, however, very close to the (Sn) + η–two-phase field; therefore the amount of T4 phase is very low and could not be detected by XRD.

### DTA data

**Fig 2:**
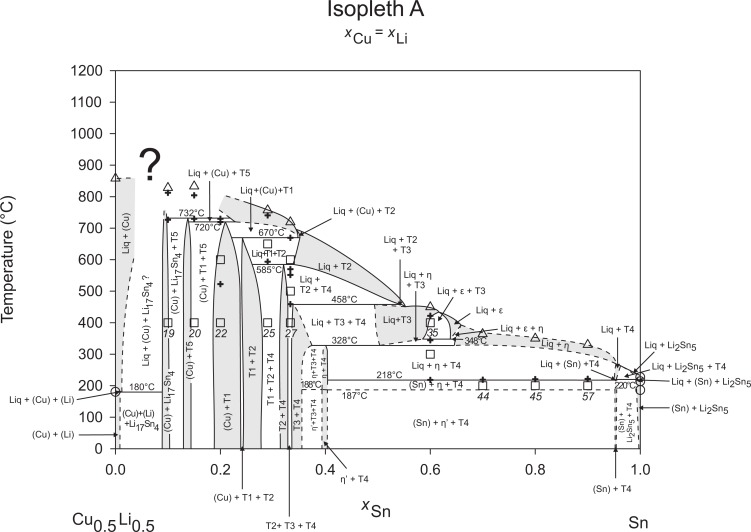
Isopleth A.

**Fig 3:**
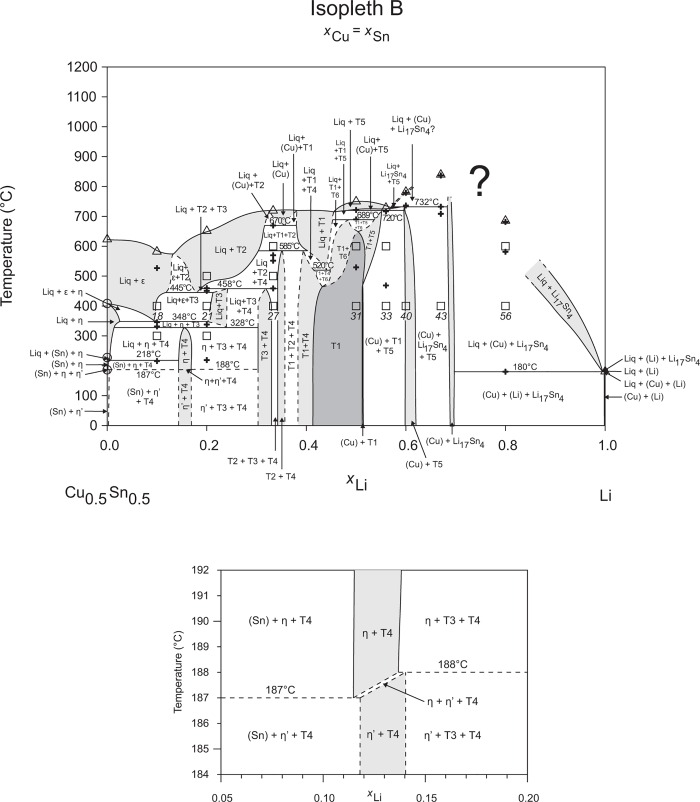
Isopleth B including section from *x*_Li_ = 0.05–0.20 / *T* = 184–192°C.

**Fig 4:**
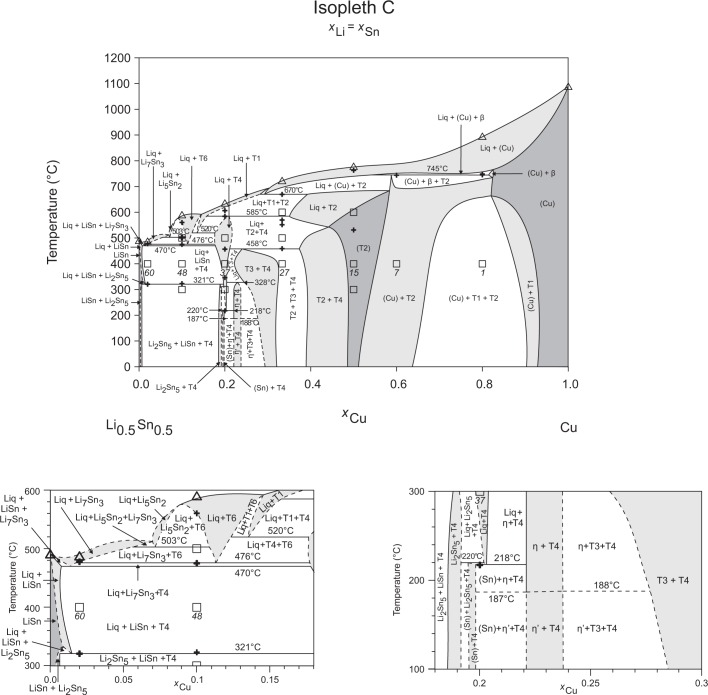
Isopleth C including both sections from *x*_Cu_ = 0–0.18 / *T* = 300–600°C and *x*_Cu_ = 0.18–0.30 / *T* = 100–300°C.

**Fig 5:**
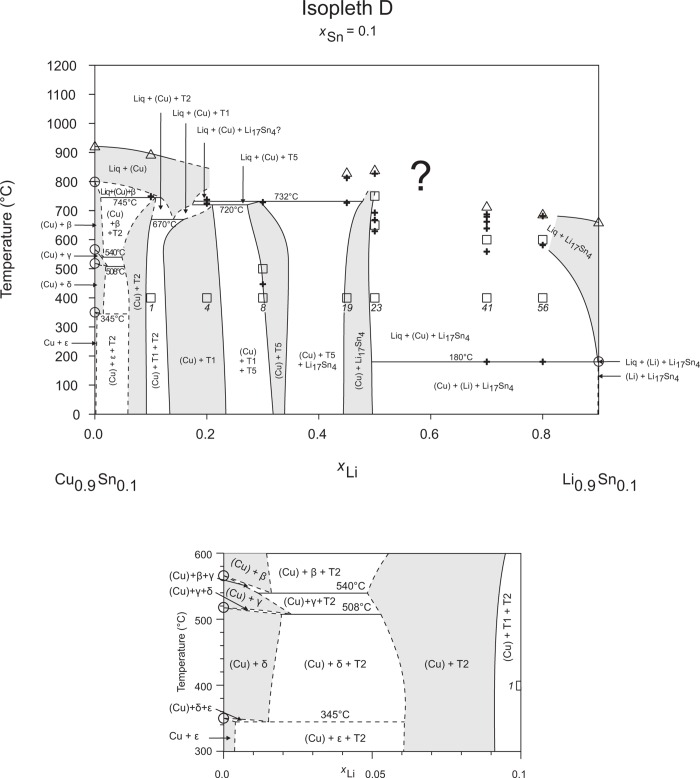
Isopleth D including section from *x*_Li_ = 0–0.10 / *T* = 300–600°C.

**Fig 6:**
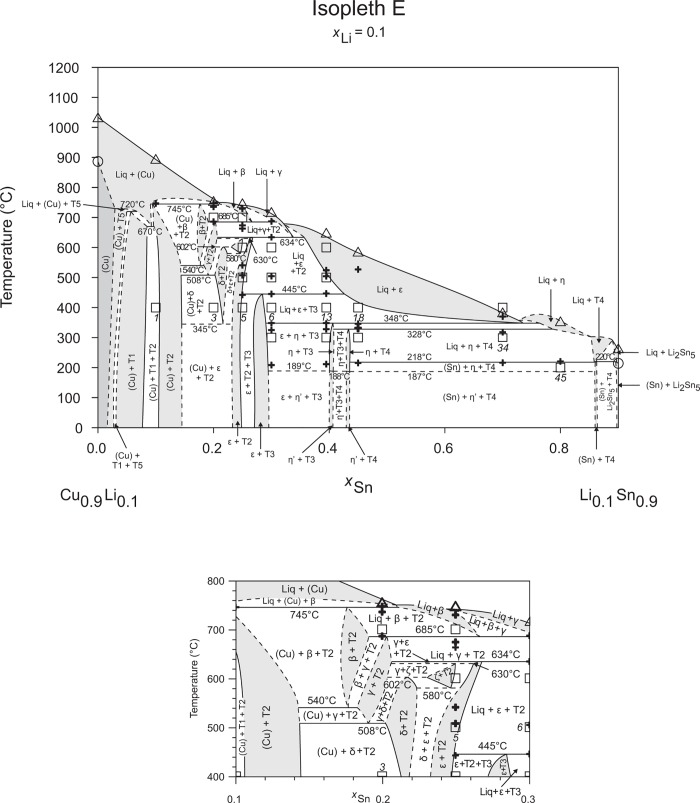
Isopleth E including section from *x*_Sn_ = 0.10–0.30 / *T* = 400–800°C.

**Fig 7:**
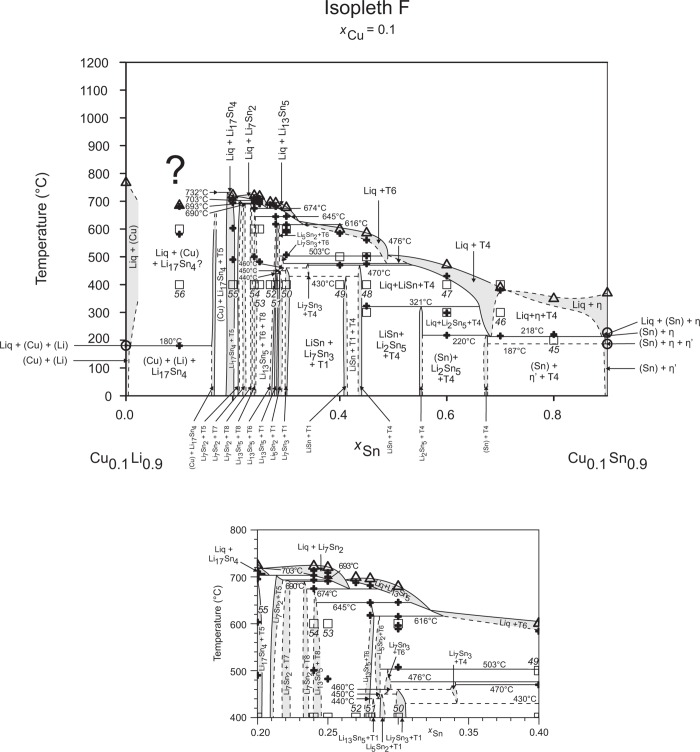
Isopleth F including section from *x*_Sn_ = 0.20–0.40 / *T* = 400–800°C.

**Fig 8:**
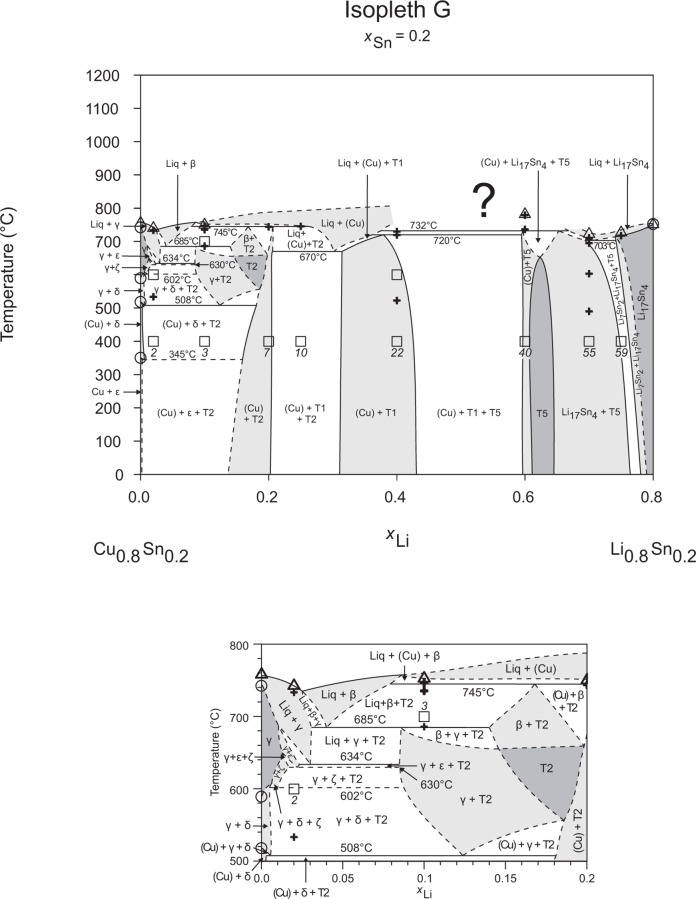
Isopleth G including section from *x*_Li_ = 0–0.20 / *T* = 500–800°C.

**Fig 9:**
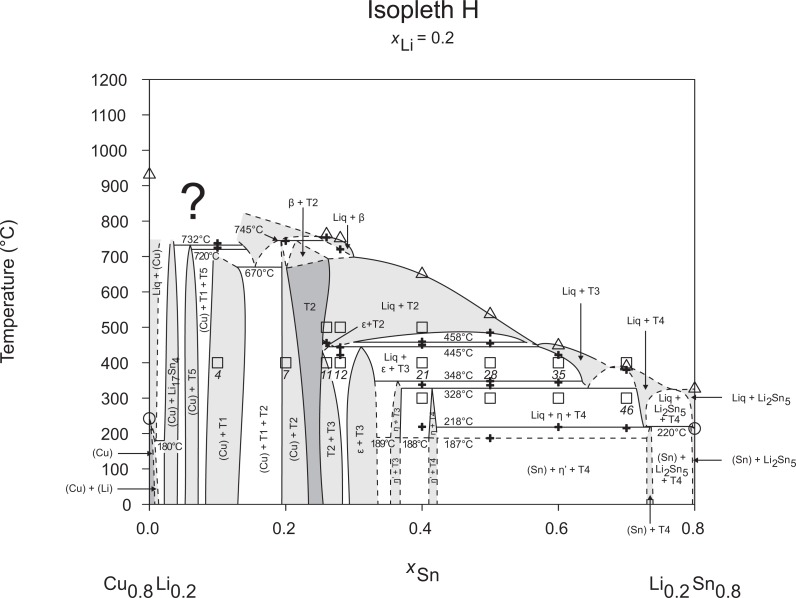
Isopleth H.

**Fig 10:**
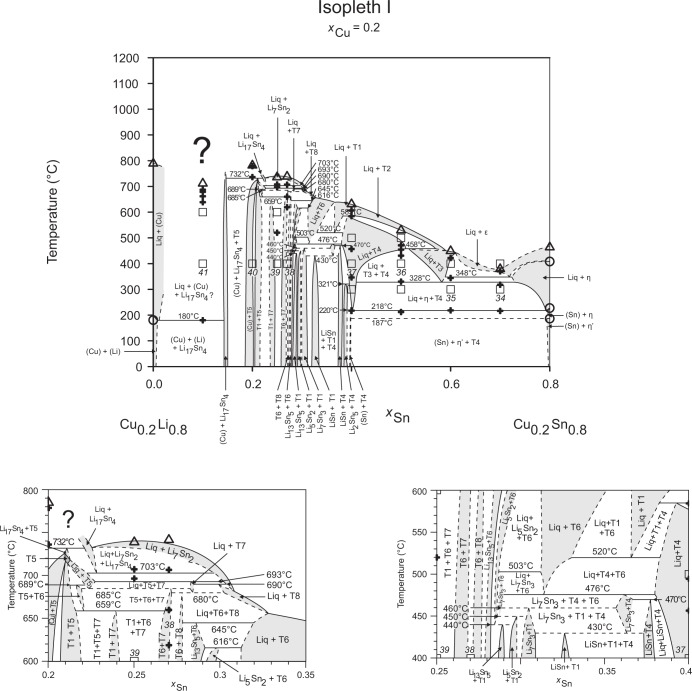
Isopleth I including sections from *x*_Sn_ = 0.20–0.35 / *T* = 600–800°C and *x*_Sn_ = 0.25–0.40 / *T* = 400–600°C.

**Fig 11:**
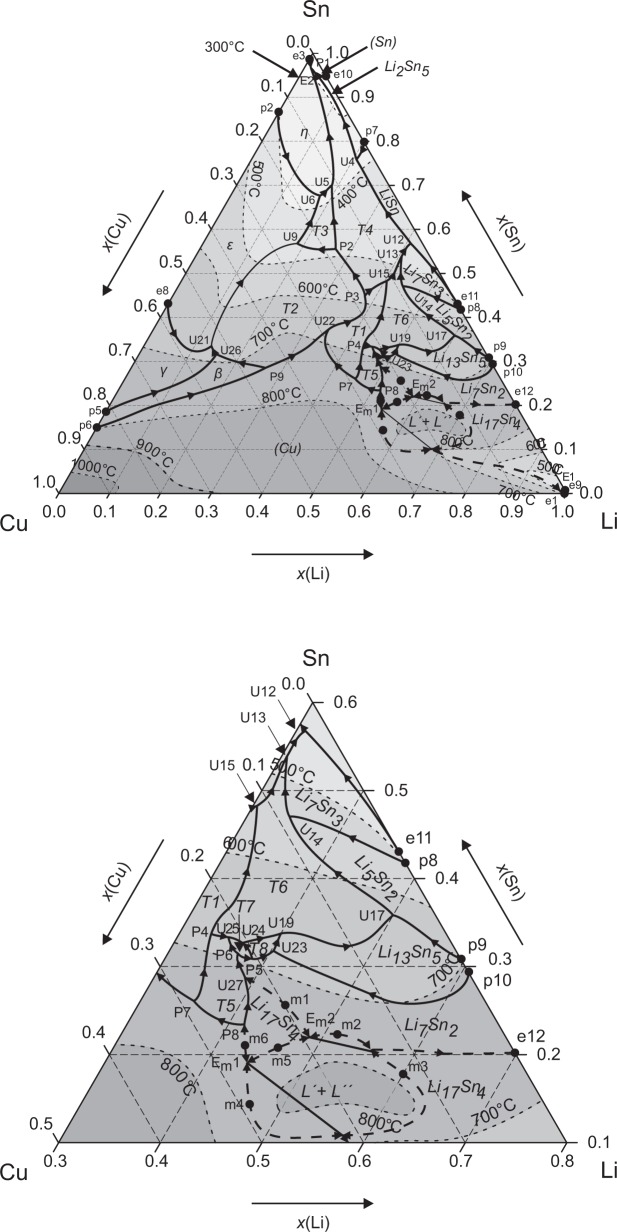
Liquidus projection including section of Li-rich corner.

The liquidus temperatures (see Table 2) of the samples show a concise picture and could be unified to the construction of the isopleths, Figs 2–10, and the liquidus projection, Fig 11. They are indicated by triangle-shaped symbols in the isopleths. Depending on the Li-content the maximum temperature of our DTA runs was chosen to be at 800–900°C. In case of the samples 4, 7, 8, 10 and 22, which are located in the Cu-rich corner of the phase diagram, the alloys were not totally molten at the maximum. Five samples with very high Sn content show diffuse melting peaks (34, 44, 45, 46, 57) – an exact determination of the peak maxima was difficult or even impossible; therefore these liquidus temperatures were carefully estimated and indicated with a swung dash symbol “~” in Table 2. The liquidus curves in the corresponding isopleths were drawn as dashed lines. Thermal effects below the liquidus are indicated by cross-shaped symbols in the respective isopleths (Figs 2–10). Most of these thermal effects occurred in more than one sample and could be allocated to invariant reactions, which have been listed in Table 3. The evaluation of these reactions is also supported by the phase equilibria of samples annealed at 300, 400, 500, and 600°C, shown in Table 1 (for the corresponding isothermal sections see Ref. [6]). Some samples, however, show peaks at temperatures, which could not directly be allocated to reaction isotherms or they are at concentrations which are not covered by the reaction isotherms. Temperatures of these heat effects are written in italic in Table 2.

**Table 3:** Invariant ternary reactions: Temperatures and reactions in dotted and italic lines are approximated.

			Reaction shown in Isopleth…								
Temperature [°C]	Reaction	Type / No.	A	B	C	D	E	F	G	H	I
180	Liq → (Cu) + (Li) + Li_17_Sn_4_	E1	x	x		x		x		x	x
*187*	*η + T4 → (Sn) + η´*	*U1*	*x*	*x*	*x*		*x*	*x*		*x*	*x*
*188*	*η + T3 → η´ + T4*	*U2*	*x*	*x*	*x*		*x*			*x*	
*189*	*ε + η → η´ + T3*	*U3*					*x*			*x*	
218	Liq → (Sn) + η + T4	E2	x	x	x		x	x		x	x
220	Liq + Li_2_Sn_5_ + T4 → (Sn)	P1	x		x		x	x		x	x
321	Liq + LiSn → Li_2_Sn_5_ + T4	U4			x			x			x
328	Liq + T3 → η + T4	U5	x	x	x		x			x	x
*345*	*δ → (Cu) + ε + T2*	*E3*				*x*	*x*		*x*		
348	Liq + ε → η + T3	U6	x	x			x			x	x
*430*	*Li*_*7*_*Sn*_*3*_ *+ T4 → LiSn + T1*	*U7*						*x*			*x*
*440*	*Li*_*5*_*Sn*_*2*_ *+ T6 → Li*_*13*_*Sn*_*5*_ *+ T1*	*U8*						*x*			*x*
445	Liq + T2 → ε + T3	U9		x			x			x	
*450*	*Li*_*7*_*Sn*_*3*_ *+ T6 → Li*_*5*_*Sn*_*2*_ *+ T1*	*U10*						*x*			*x*
458	Liq + T2 + T4 → T3	P2	x	x	x					x	x
*460*	*T4 + T6 → Li*_*7*_*Sn*_*3*_ *+ T1*	*U11*						*x*			*x*
470	Liq + Li_7_Sn_3_ → LiSn + T4	U12			x			x			x
476	Liq + T6 → Li_7_Sn_3_ + T4	U13			x			x			x
503	Liq + Li_5_Sn_2_ → Li_7_Sn_3_ + T6	U14			x			x			x
508	γ → (Cu) + δ + T2	E4				x	x		x		
520	Liq + T1 → T4 + T6	U15		x	x						x
540	β → (Cu) + γ + T2	E5				x	x				
*580*	*ζ → δ + ε + T2*	*E6*					*x*				
585	Liq + T1 + T2 → T4	P3	x	x	x						x
*602*	*γ + ζ → δ + T2*	*U16*					*x*		*x*		
616	Liq + Li_13_Sn_5_ → Li_5_Sn_2_ + T6	U17						x			x
*630*	*γ + ε → ζ + T2*	*U18*					*x*		*x*		
634	Liq + γ → ε + T2	U19					x		x		
645	Liq + T8 → Li_13_Sn_5_ + T6	U20						x			x
659	T5 + T6 → T1 + T7	U21									x
670	Liq + (Cu) → T1 + T2	U22	x	x	x	x	x		x	x	
674	Liq + Li_7_Sn_2_ → Li_13_Sn_5_ + T8	U23						x			
*680*	*Liq + T7 → T6 + T8*	*U24*									*x*
*685*	*Liq + T5 → T6 + T7*	*U25*									*x*
685	Liq + β → γ + T2	U26					x		x		
689	Liq + T1 + T5 → T6	P4		x							x
690	Liq + Li_7_Sn_2_ + T7 → T8	P5						x			x
693	Liq + Li_7_Sn_2_ + T5 → T7	P6						x			x
703	Liq + Li_17_Sn_4_ → Li_7_Sn_2_ + T5	U27						x	x		x
720	Liq + (Cu) + T5 → T1	P7	x	x		x	x		x	x	
732	Liq + (Cu) + Li_17_Sn_4_ → T5	P8	x	x		x		x	x	x	x
745	Liq + (Cu) + β → T2	P9			x	x	x		x	x	
*700–800*	*Liq´* → *Liq´´ + (Cu) + Li*_*17*_*Sn*_*4*_	*Em1*									
*753–800*	*Liq´* → *Liq´´ + Li*_*7*_*Sn*_*2*_ *+ Li*_*17*_*Sn*_*4*_	*Em2*									

### Isopleths

Figs 2–10 represent the nine isopleths A to I where additional information is given as follows:

- Square symbols illustrate sample compositions at annealing temperatures and correspond to the data given in Table 1.
- Cross-shaped symbols indicate invariant or non-invariant heat effects from DTA signals below the liquidus temperature.
- Triangle-shaped symbols represent liquidus temperatures.
- Circle-shaped symbols indicate binary reacting temperatures from literature.
- Italic numbers designate the sample number according to Tables 1 and 2.

In a first step of construction all available transformation temperatures [8–10, 19] have been plotted along the respective sections. The Cu_2_Li_3_ phase, which was postulated by Gąsior et al. [20], was neglected as discussed in detail by Li et al. [8]. A summary of employed invariant binary reactions is given in Table 4. In a second step of construction, phase equilibria from the isothermal sections at 300, 400, 500, 600°C [6] and at other temperatures (for all cases see phase analysis by XRD in Table 1) have been included. Due to the lack of microprobe chemical analysis of equilibrium phase compositions, phase field limits had to be estimated. Table 5 summarizes all three-phase equilibria and samples which can be allocated to the associated phase fields. Symbol “x” in the Table indicates that the presence of the phase field at the respective temperature was not directly proven by experiments. However, an assignment was possible regarding adjacent phase fields and results of same alloy compositions annealed at nearby temperatures.

**Table 4:** Invariant reactions of the binary subsystems.

	Invariant reactions	Reaction type	Temperature [°C]	Ref.
Unary phases	Liq → (βLi)	c1	180.6	[22]
	Liq → (βSn)	c2	231.9681	[22]
	Liq → (Cu)	c3	1084.87	[23]
Cu-Li	Liq → (Cu) + (Li)	e1	180.5	[11]
Cu-Sn	η → (Sn) + η´	e2	186	[23]
	ε + η → η´	p1	189.1	[23]
	Liq → (Sn) + η	e3	227	[23]
	δ → (Cu) + ε	e4	350	[23]
	Liq + ε → η	p2	408	[9]
	γ → (Cu) + δ	e5	518	[9]
	ζ → δ + ε	e7	589	[9]
	β → (Cu) + γ	e6	566	[9]
	γ + ζ → δ	p3	603	[9]
	γ + ε → ζ	p4	641	[9]
	γ → Liq + ε	e8	649	[9]
	ε → γ	c4	676	[23]
	Liq + β → γ	p5	758	[9]
	Liq + (Cu) → β	p6	798	[9]
Li-Sn	Liq → (βLi) + Li_17_Sn_4_	e9	180.6	[22]
	Liq → Li_2_Sn_5_ + (βSn)	e10	214	[22]
	Liq + LiSn → Li_2_Sn_5_	p7	327	[10]
	Liq → LiSn	c5	486	[10]
	Liq → Li_7_Sn_3_ + LiSn	e11	473	[10]
	Liq + Li_5_Sn_2_ → Li_7_Sn_3_	p8	509	[10]
	Liq + Li_13_Sn_5_ → Li_5_Sn_2_	p9	698	[10]
	Liq + Li_7_Sn_2_ → Li_13_Sn_5_	p10	724	[10]
	Liq → Li_17_Sn_4_ + Li_7_Sn_2_	e12	752	[10]
	Liq → Li_17_Sn_4_	c6	758	[10]
	Liq → Li_7_Sn_2_	c7	779	[10]

**Table 5:** Three-phase equilibria in Cu-Li-Sn directly derived from experiments.

Phases in three-phase field	Corresponding samples at annealing temperatures				
	300°C	400°C	500°C	600°C	Other Temp.
Liq´ + (Cu) + Li_17_Sn_4_	x	23, 41, 56	x	41, 56	23 (650 / 750°C)
Liq´´ + (Cu) + Li_17_Sn_4_					
Liq + β + T2					3, 5 (700°C)
Liq + ε + T2			3, 13, 14	6	
Liq + ε + T3		6, 13, 14, 18, 21, 28, 35			
Liq + ε + η		x			
Liq + η + T4	18, 28, 34, 35, 36, 46				
Liq + Li_13_Sn_5_ + T8					51 (650°C)
Liq + Li_2_Sn_5_ + T4	47				
Liq + Li_5_Sn_2_ + T6				50	
Liq + Li_7_Sn_3_ + T6			48, 49		
Liq + LiSn + T4		48, 60			
Liq + T1 + T2				27	25 (650°C)
Liq + T1+ T6				x	
Liq + T2 + T4			24, 27, 36		
Liq + T3 + T4		36			
(Cu) + δ + T2		2, 3	x		
(Cu) + Li_17_Sn_4_ + T5	x	32, 43	x	x	
(Cu) + T1 + T2	x	1, 10	x	x	
(Cu) + T1 + T5	x	8, 20, 30, 33	x	x	
(Sn) + η + T4					44, 45, 57 (200°C)
γ + δ + T2				2	
δ + ζ + T2				x	
ε + ζ + T2				5	
ε + η + T3	6, 13, 14				
ε + T2 + T3	x	5, 9, 12			
η + T3 + T4	21				
Li_13_Sn_5_ + T1 + T6		51			
Li_13_Sn_5_ + T6 + T8		52, 53		53	
Li_5_Sn_2_ + Li_13_Sn_5_ + T1	x	x			
Li_5_Sn_2_ + Li_7_Sn_3_ + T1	x	x			
Li_7_Sn_2_ + Li_13_Sn_5_ + T8		54		54	
Li_7_Sn_2_ + Li_17_Sn_4_ + T5	x	x	x	x	
LiSn + Li_2_Sn_5_ + T4	48, 60				
LiSn + Li_7_Sn_3_ + T1	x	49			
LiSn + T1 + T4	x	x			
T1 + T2 + T4	x	25	x		
T1 + T5 + T7				x	
T1 + T6 + T7	x	39		39	
T2 + T3 + T4	x	16, 24, 27			

Heat effects of neighbouring samples at similar temperatures were connected with horizontal lines in the isopleth schemes and attributed to invariant reactions. Resulting single-, two- and three-phase fields were constructed strictly respecting the rule of Landau and Palatnik [21]. The single-phase fields in Figs 2–10 are illustrated in dark grey, two-phase fields are shown light grey and three-phase fields are presented in white. All ternary phases were found to be formed peritectically. The peritectic formation temperatures of phases T1-T6 are 720, 745, 458, 585, 732 and 689°C, respectively (see Table 3). The peritectic formation temperatures of phases T7 and T8 were estimated to be at 693 and 690°C. In a third step of construction it was verified that each isopleth adapts to other ones along their intersections. In total there are seven intersections involving three isopleths and twelve intersections involving two isopleths; Fig 1. To keep the amount of prepared alloys within reasonable limits some phase fields or invariant reactions had to be assumed for the construction of isopleths. They are not validated by experiments and therefore drawn as dotted lines in Figs 2–10 and written in italic in Tables 3. Especially in the Li-rich corner (see isopleths F and I), where five binary Li-Sn phases and at least three ternary phases exist next to each other, phase fields must be very narrow (< 1 at. %) and reliable experimental investigations are impossible regarding the inaccuracy of sample compositions; see section “Sample preparation” and Ref. [6]. The allocation of respective phase fields in this region is estimated and phase transformations could be solely adumbrated by vicinal data and thermodynamic rules. In the Cu-rich corner, several binary Cu-Sn phases make isopleths E and G more complex, especially between 500°C and liquidus. Generally, in order to show complex regions of isopleths with a very close sequence of phase fields they are magnified in some cases in Figs 3–10. For isopleths crossing the liquid miscibility gap mentioned above (all but C and E), the respective phase equilibria could not be established based on our experimental data. These regions are indicated by a question mark.

### Liquidus projection

The liquidus projection of the Cu-Li-Sn system is presented in Fig 11 and is in consistence with all isopleths and liquidus temperatures from DTA and our XRD results. A major experimental limitation is the lack of metallography and phase selective chemical analyses by EPMA due to the instability against air and moisture and the low atomic number of lithium. Therefore it was, *e*.*g*. not possible to directly identify fields of primary crystallisation from furnace cooled samples.

The primary crystallisation field of (Cu), which is the highest melting phase dominates approximately one third of the liquidus surface, followed by T2 phase, which holds the highest peritectic formation temperature (745°C) of all ternary phases. The primary crystallisation of T1, T3 and T4 is suggested to be in the direct surrounding. Comparably large primary crystallisation fields are estimated for ε and γ – and a narrower phase field for β, which extends far into the centre of the phase diagram. In the Sn-rich corner, the dominant primary crystallisation field is that one of the η-phase. The Cu-Sn phases δ, ζ and η´ are formed peritectoidically without involving the liquid phase (*cf*. Table 4) and are therefore the only phases do not show up in the liquidus projection. The ternary phases T5-T8 and most of the binary Li-Sn phases have their primary crystallisation field within the compositional triangle Cu_0.4_Li_0.4_Sn_0.2_ – Li_0.8_Sn_0.2_ – Li_0.4_Sn_0.6_ (see magnified section in Fig 11). As it was difficult to synthesize samples containing only one of the two phases T7 and T8 we assumed only very small regions of primary crystallisation.

The liquidus projection in Fig 11 shows as well the liquidus isotherms based on our DTA results. It can be observed that the liquidus temperatures mostly descend from the boundary binary systems. Two liquidus valleys, descending towards the Li-corner and towards the Sn-corner, respectively separate the ternary system. Liquidus temperatures of various samples in the Li-rich part clearly indicate a ternary maximum at about 850°C. There is no experimental indication for the existence of a congruently melting ternary compound. This is supported by the high compound-forming tendency in the binary Li-Sn system [10, 24] compared to Cu-Sn [25] and Cu-Li [8]. Consequently, this maximum can only be caused by a ternary liquid miscibility gap. This assumption is supported by the assessment of the liquid phase in Li et al. [8]. Similar systems, which show a metastable liquid miscibility gap in one of the constituent binaries and a stable one in the ternary, are C-Cu-Fe [26, 27] or Al-Cu-Sn [28]. In our case, the fields of primary crystallization of Cu, Li_17_Sn_4_ and Li_7_Sn_2_ are very close to each other. Thus, we assume an extension of the liquid immiscibility over these three primary crystallization fields. This is similar to the Al-Mg-Sc system [29], which however shows no metastable binary miscibility gap. Accordingly, two ternary monotectic reactions (Em1 and Em2) are proposed based on a wide maximum of the liquidus surface in the Li-rich corner (around Cu_0.2_Li_0.65_Sn_0.15_). Each ternary monotectic reaction involves two four-phase equilibria at different liquid compositions, which are connected by a tie line indicated as solid thin line in Fig 11. Both reactions involve four maxima (m2 – m5) and the solid phases (Cu), Li_17_Sn_4,_ and Li_7_Sn_2_. It is noteworthy that for the phase Li_17_Sn_4_ two primary crystallisation fields exist. Because the existence and localisation of both reactions are only estimated, the reactions are drawn with dotted lines. Two further maxima (m1 and m6) have to be established to connect the monotectic reactions to adjacent invariant reactions (P8 and U27).

### Reaction scheme

**Fig 12:**
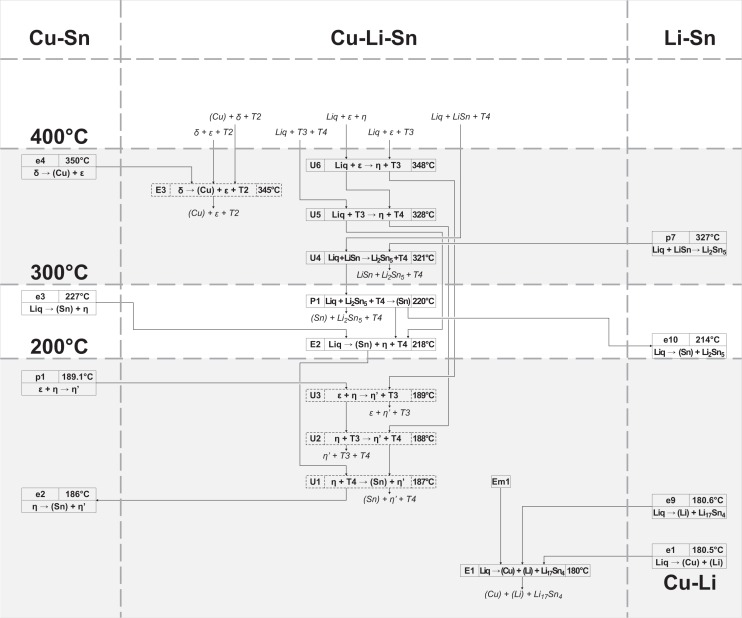
Reaction scheme *T* < 400°C.

**Fig 13:**
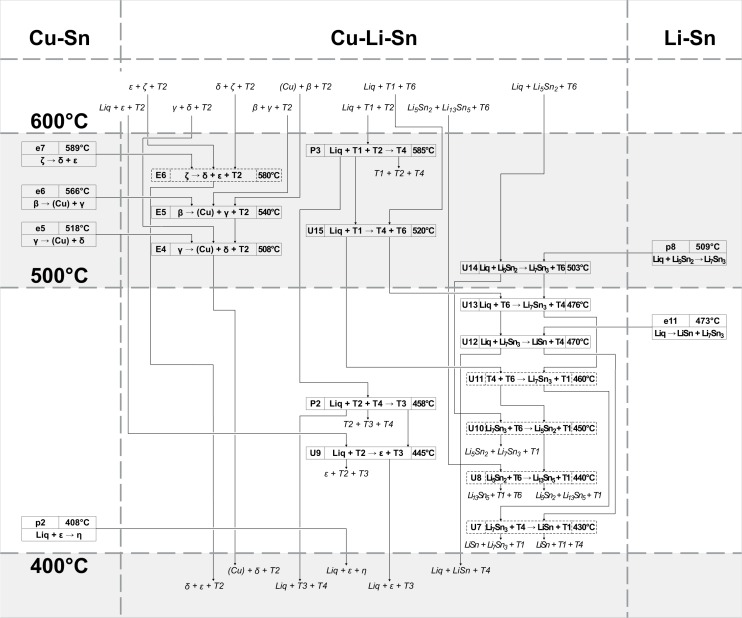
Reaction scheme *T* = 400–600°C.

**Fig 14:**
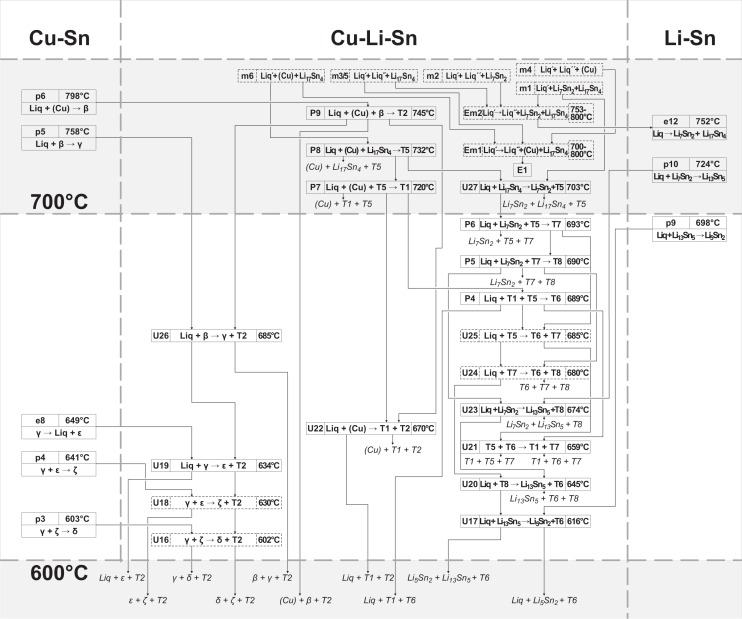
Reaction scheme *T* > 600°C.

The complete reaction scheme of the Cu-Li-Sn system is shown in three temperature intervals (Figs 12, 13 and 14 illustrate reaction schemes until 400°C, from 400–600°C and higher than 600°C, respectively). It involves 22 binary (Table 4) and 44 ternary reactions (Table 3), where 14 out of them had to be assumed due to uncertain or missing experimental data. Three unary temperatures for the pure elements, three congruent melting points (compounds Li_17_Sn_4_, Li_7_Sn_2_ and LiSn) and the congruent formation of ε from γ phase are listed in Table 4 but not shown in Figs 12–14. Two suggested ternary monotectic reactions (Em1: Liq´ → Liq´´ + (Cu) + Li_17_Sn_4_ and Em2: Liq´ → Liq´´ + Li_7_Sn_2_ + Li_17_Sn_4_) are located between approximately 700 and 800°C and involve two different liquid phases Liq´ and Liq´´. Ternary phase fields connected to the maximum points m1-m6 end up in Em1, Em2, P8, and U27. The high temperature part of the reaction scheme above 700°C (Fig 14) is dominated by a sequence of peritectic formations of ternary compounds, involving liquid, (Cu) and a fourth phase (745°C: Liq + (Cu) + β → T2; 732°C: Liq + (Cu) + Li_17_Sn_4_ → T5; 720°C: Liq + (Cu) + T5 → T1). Phases T6, T7 and T8 are formed at somewhat lower temperatures involving Li-rich phases (693°C: Liq + Li_7_Sn_2_ + T5 → T7; 690°C: Liq + Li_7_Sn_2_ + T7 → T8; 689°C: Liq + T1 + T5 → T6). An U-type reaction in the Li-rich region at 703°C includes the liquid phase, the congruent melting phases Li_17_Sn_4_ and Li_7_Sn_2_ and the Li-rich ternary phase T5 (U27: Liq + Li_17_Sn_4_ → Li_7_Sn_2_ + T5). A cascade of further U-type reactions, connecting binary Li-Sn compounds and Li-rich ternary compounds T1, T5, T6, T7 and T8 (U17, 20, 21, 23), follows; corresponding heat effects can be compared with Figs 7A and 10A (magnified sections of isopleths F and I). U24 (680°C: Liq + T7 → T6 + T8) and U25 (685°C: Liq + T5 → T6 + T7) are not based on measured DTA effects and had to be estimated from the liquidus projection (Fig 11). A central reaction, which is well identified by means of XRD and DTA, is U22 (670°C: Liq + (Cu) → T1 + T2). At temperatures up to 670°C, three-phase fields are separated by the dominant two-phase field T1 + T2 (compare isothermal section in [6]); above 670°C, this two-phase field connects (Cu) and Sn-rich liquid. The reaction U22 is present in most isopleths (A-E, G, and H) and maintains an interesting shape of the Liq + (Cu) two-phase region (compare isopleths D, E, H). At 585°C the phase T4 is formed from Liq, T1 and T2 (P3: Liq + T1 + T2 → T4), which enables several following reactions with adjacent phases T1, T6, Li_7_Sn_3_, Li_5_Sn_2_ and LiSn (U12 – U15). The phase T1 occurs in samples 50 and 51 at 400°C together with T6. The latter one is also present in samples with increased Li-concentrations (#52 and #53) at 600°C. This requires further reactions between 400 and 500°C; U7, U8, U10 and U11 at 430, 440, 450 and 460°C, respectively. They have been included into the reaction scheme even so they are only tentative and therefore drawn with dashed lines (as well as in isopleths F and I). The lowest ternary phase formation temperature is that one of phase T3, which is formed from liquid, T2 and T4 at 458°C. It is involved in three U-type reactions with binary Cu-Sn phases ε and / or η (U9 at 445°C: Liq + T2 → ε + T3; U6 at 348°C: Liq + ε → η + T3; U5 at 328°C: Liq + T3 → η + T4). The Sn-rich corner is dominated by liquid and T4 phase. Three reactions follow from 321°C (U4: Liq + LiSn → Li_2_Sn_5_ + T4) to 220°C (P1: Liq + Li_2_Sn_5_ + T4 → (Sn)) and finally to 218°C (E2: Liq → (Sn) + η + T4). Four reactions take place at temperatures < 200°C (E1, U1-U3), but except for E1 (180°C: Liq → (Cu) + (Li) + Li_17_Sn_4_) no evident heat effect could be found experimentally. Therefore reactions U1-U3 were estimated based on the vicinal binary reactions e2 (186°C: η → (Sn) + η´) and p1 (189.1°C: ε + η → η´) and are indicated using dashed lines. The Cu-rich side is extrapolated from the binary Cu-Sn system into the ternary system. The phase T2 is the dominating one in this region; therefore most reactions including liquid and Cu-Sn phases are connected to T2 (E3-E6, U16, U18 and U26). These ternary reactions occur at temperatures close to the respective binary reactions.

## Conclusions and Outlook

Reactions and reaction temperatures between two liquid phases, three unary phases, 14 binary phases and 8 ternary phases have been widely clarified by combination of XRD and DTA data. An iterative development of isopleths, isotherms and a liquidus projection, under the consideration of most DTA and XRD results, leads to a consistent description of the phase diagram. The present phase diagram, which is illustrated by nine isopleths, a liquidus projection and a reaction scheme, includes 113 three-phase regions, which are related to 44 ternary invariant reactions. In some parts of the phase diagram, namely in the vicinity of Li-rich binary Li-Sn phases, in some regions close to the Cu-rich binary Cu-Sn phases and at temperatures above 750 and below 200°C, no clear experimental data were available. Thus assumptions of phase equilibria and reaction temperatures based on adjacent samples had to be made which still require further clarification. In addition, the existence of the two monotectic ternary reactions Em1 and Em2 should be proved in further investigations. The knowledge of the phase diagram offers the possibility to prepare alloys with predetermined phase composition and microstructure. It is also a valuable reference for a calculated phase diagram, which is usually based on an optimization of thermodynamic data and performed with the CALPHAD approach [30]. An optimization based on this phase diagram and experimental thermochemical data allows the calculation of physicochemical properties for certain regions of the phase diagram, *e*.*g*. open circuit potentials. These inputs are necessary for a tailored design of materials for application in Li-ion batteries and legitimate fundamental research in the context of applied science.
